# The impact of discharge letter content on unplanned hospital readmissions within 30 and 90 days in older adults with chronic illness – a mixed methods study

**DOI:** 10.1186/s12877-024-05172-1

**Published:** 2024-07-10

**Authors:** Igor Adelsjö, Elin C. Lehnbom, Amanda Hellström, Lina Nilsson, Maria Flink, Mirjam Ekstedt

**Affiliations:** 1 Department of Health and Caring Sciences, Faculty of Health and Life Sciences, Linnaeus University, 39182 Kalmar, Sweden; 2 Department of Pharmacy, Faculty of Health Sciences, UiT The Arctic University of Norway, Tromsø, Norway; 3 Department of Medicine and Optometry, Faculty of Health and Life Sciences, eHealth Institute, Linnaeus University, Kalmar, Sweden; 4 Department of Neurobiology, Care Sciences and Society, Karolinska Institutet, Stockholm, Sweden; 5 Learning, Informatics, Management and Ethics (LIME), Karolinska Institutet, Stockholm, Sweden

**Keywords:** Chronic obstructive pulmonary disease, Communication, Congestive heart failure, Hospital discharge, Medication therapy management, Self-management, Polypharmacy

## Abstract

**Background:**

Care transitions are high-risk processes, especially for people with complex or chronic illness. Discharge letters are an opportunity to provide written information to improve patients’ self-management after discharge. The aim of this study is to determine the impact of discharge letter content on unplanned hospital readmissions and self-rated quality of care transitions among patients 60 years of age or older with chronic illness.

**Methods:**

The study had a convergent mixed methods design. Patients with chronic obstructive pulmonary disease or congestive heart failure were recruited from two hospitals in Region Stockholm if they were living at home and Swedish-speaking. Patients with dementia or cognitive impairment, or a “do not resuscitate” statement in their medical record were excluded. Discharge letters from 136 patients recruited to a randomised controlled trial were coded using an assessment matrix and deductive content analysis. The assessment matrix was based on a literature review performed to identify key elements in discharge letters that facilitate a safe care transition to home. The coded key elements were transformed into a quantitative variable of “SAFE-D score”. Bivariate correlations between SAFE-D score and quality of care transition as well as unplanned readmissions within 30 and 90 days were calculated. Lastly, a multivariable Cox proportional hazards model was used to investigate associations between SAFE-D score and time to readmission.

**Results:**

All discharge letters contained at least five of eleven key elements. In less than two per cent of the discharge letters, all eleven key elements were present. Neither SAFE-D score, nor single key elements correlated with 30-day or 90-day readmission rate. SAFE-D score was not associated with time to readmission when adjusted for a range of patient characteristics and self-rated quality of care transitions.

**Conclusions:**

While written summaries play a role, they may not be sufficient on their own to ensure safe care transitions and effective self-care management post-discharge.

**Trial registration:**

Clinical Trials. giv, NCT02823795, 01/09/2016.

**Supplementary Information:**

The online version contains supplementary material available at 10.1186/s12877-024-05172-1.

## Background

As people age, they often experience reduced physical function, mobility limitations, cognitive impairments, and social isolation [1, 2]. Additionally, the higher prevalence of multiple health conditions (multimorbidity) in older adults poses challenges during transitions between caregivers [3, 4]. These challenges may involve understanding and managing self-care, medications, and coordination among different caregivers [5, 6]. Patients’ understanding of the hospitalization, often provided as a written discharge letter to the patient, may therefore be important features to diminish the risks of care transitions. Currently care transitions are high-risk processes, often resulting in adverse events or preventable readmissions [7], especially for older people with complex or chronic illnesses. Approximately one in five patients experience adverse events leading to human suffering and increased use of healthcare resources [8, 9]. The thirty-day readmission rate for older patients with chronic illnesses has been demonstrated to range up to 20% for all causes [10, 11]. Comorbidity, excessive polypharmacy, and a length of stay of more than five days at the initial admission were factors that increased the risk of readmission in a recent Swedish study [12]. Almost 40% of the readmissions within 30 days were classified as possibly medication-related [13]. Unfortunately, many patients are not prepared for self-management activities at home and leave the hospital with an incomplete understanding of their diagnoses, medication changes and the need for self-management activities [5, 6]. Furthermore, the decision to discharge a patient from hospital is often made hastily, on the day of discharge, and the time for discussion of these topics is limited [6].

To improve the quality of care transitions, intervention studies where clinical pharmacists, nurses and other healthcare professionals contributed to care transitions have been carried out, though with inconsistent results. For example, a multifaceted intervention with clinical pharmacists reduced 180-day readmission rates [14] and an educational programme led by nurses reduced 12-month readmission rates [15]. In patients after acute myocardial infarction (AMI), an increased hospital utilization of the standard discharge contract—emphasizing guideline-based medications, lifestyle modification, and follow-up planning—was significantly linked to decreased 1-year mortality [16]. However, in other studies, no difference [17] or even increased readmission rates were observed in the intervention group compared with the control group [18]. In a study examining the impact of standardized electronic discharge instructions with embedded computerized medication reconciliation, researchers anticipated a reduction in post-discharge hospital utilization. However, they were surprised to find a slight but statistically significant increase in 30-day readmission rates [19]. In a separate study [7] although no significant link was observed between discharge documentation and readmission, researchers did identify connections between readmission rates and follow-up arrangements after discharge, as well as the number of medications prescribed. This raises the question of whether improving discharge quality should target metrics beyond mere documentation. Systematic reviews have identified that interventions with multifaced components, including support of patients’ self-management capacity, are the most effective in reducing readmission rates [20, 21].

Discharge information about medication regimens, lifestyle factors and how to handle symptoms after discharge is essential for patients to develop the capacity for self-management at home [22]. Discharge information is communicated to outpatient healthcare professionals in a discharge summary and to patients in a discharge letter written in lay language [23]. This means that the information must be adapted to the recipient's experience, linguistic background, and other individual conditions to ensure as far as possible that the recipient understands the content and significance of the information provided [24] Discharge letters are thus an opportunity to support patient activation. This is usually assessed based on patients’ self-reported knowledge, skills, and confidence to manage their symptoms, disabilities and complex treatments at home [25]. The contents of discharge letters to patients have been categorised and evaluated in previous studies [26, 27]. Information that has been identified as particularly useful for patients after discharge from inpatient hospital care includes “Reason for admission”, “Procedures performed during admission”, “Medication list” and “Advise about anticipated problems” [27]. To improve the quality of the discharge process and reduce readmission rates for older adults with chronic illness, there is a need to further explore the contents of discharge letters and examine if they support patients in their capacity for self-management after discharge.

### Aim

To determine the impact of discharge letter content on unplanned hospital readmissions within 30 and 90 days, and to identify correlations between discharge letter content and quality of care transitions in patients 60 years of age or older with chronic illness.

## Methods

### Design

This study was a convergent mixed methods design [28] using discharge letters and a selection of quantitative data collected in a randomised controlled trial (RCT) of a care transition intervention (sPATH). The sPATH intervention was developed to support patient activation after hospital discharge and reduce 90-day readmission rates for patients with chronic illness. The intervention, conducted at two hospitals, involved motivational interviewing to enhance patient self-management, compared to standard care. The intervention has been described in detail elsewhere [29].

### Setting and participants

In brief, patients with chronic obstructive pulmonary disease or congestive heart failure admitted to one of two hospitals in Region Stockholm, Sweden, were invited to participate in the RCT if they were 18 years or older and living in their own home. Exclusion criteria were diagnosis of dementia or mild cognitive impairment, need for an interpreter, and patients with a statement of “do not resuscitate” in their medical record. Patients randomised to the intervention group met a patient activation coach during five sessions over the course of four weeks post-discharge to support self-management and motivate them for activation at home. Patients in the control group received care as usual, which did not include any counselling or support of their self-management needs or activation post-discharge. Only patients 60 years of age or older who had received a discharge letter were included in this study. Out of the 207 patients initially included in the RCT, 53 did not receive a discharge letter at discharge, and 18 patients younger than 60 years of age were excluded. In total, 136 patients aged above 60 years (70 men, 66 women; mean age 76.2 years) from either the intervention group (*n* = 71) or the control group (*n* = 65) were included in this study.

### Measures

#### Outcome measures

Data regarding healthcare consumption included all-cause unplanned hospital admission within 180 days before index admission, time to first unplanned readmission at 90 days from index admission, 30-day and 90-day unplanned readmission rates, and length of stay during index admission.

#### Patient characteristics at baseline

To assess patient characteristics at baseline, demographic data on age, sex, educational level, living arrangements, income level and country of birth was gathered. Clinical data on multimorbidity, i.e., number of diagnoses (identified using codes in the International Classification of Diseases, 10th revision, ICD-10) at the time of discharge as well as the Charlson Comorbidity Index were included, as were prescribed medications dispensed from pharmacies within 180 days before index admission. Further, the baseline measures of patients’ *self-rated knowledge, skills, and confidence in self-management* were included and assessed with the Swedish translation of the 13-item Patient Activation Measure (PAM) [25, 30]. The raw scores of PAM can be converted into four activation levels on a scale ranging from 0 to 100, where higher scores indicate greater patient activation. Level 1 (≤ 47.0) indicates not believing activation to be important, level 2 (47.1–55.1) signifies a lack of knowledge and confidence to take action, level 3 (55.2–67.0) indicates beginning to take action and level 4 (≥ 67.1) implies taking action [25].

*Self-rated quality of care transitions* was measured using the Swedish translation of the 3-item version (CTM-3), i.e., items 2, 9 and 13 of the original 15-item Care Transition Measure (CTM) [31, 32]. The three items are: “The hospital staff took my preferences into account in deciding what my healthcare needs would be when I left the hospital”, “When I left the hospital, I had a good understanding of the things I was responsible for in managing my health” and “When I left the hospital, I clearly understood the purpose for taking each of my medications”. Higher scores on CTM-3 have been shown to be associated with a lower risk of hospital readmission within 30 days [33].

For both the CTM-3 and PAM questionnaires, patients rate each item from 1 = “strongly disagree” to 4 = “strongly agree” or “not applicable”, and answers are recalculated to a scale of 0–100.

#### Procedures

Patient characteristics and the PAM questionnaire were collected at inclusion in the RCT, i.e., during the index admission. Patients received the CTM-3 questionnaire at discharge and were asked to fill it out at home and return it to the researchers by post within one week. Discharge letters were retrieved from the medical record after discharge. Register data of healthcare consumption were retrieved from Region Stockholm’s Register for Healthcare Encounters for the periods 365 days before and 180 days after the day of inclusion in the RCT, including prescribed medications dispensed from pharmacies within the same periods (identified using codes in the Anatomic Therapeutic Chemical classification system, ATC).

### Data analysis

Data analysis in a convergent design consists of three phases [28]. First, a matrix to assess key elements of discharge letters was developed based on a literature review, and a deductive content analysis was conducted to code the discharge letters in accordance with the matrix. Next, the coded key elements were transformed into a quantitative variable labelled “SAFE-D score” which was combined with the dataset from the RCT. Lastly, the combined data were analysed with appropriate statistical methods and presented visually in the results.

The matrix for deductive coding of discharge letters was developed based on a literature review, performed to identify key elements that should be included in discharge letters to facilitate safe care transitions. Literature searches were conducted in PubMed, Cinahl and Cochrane with the keywords “Discharge communication”, “Discharge information”, “Discharge instructions”, “Discharge letter” and “Discharge summary”. Although discharge summaries are typically clinician-directed, they may contain pertinent information that could inform the content of patient-directed discharge letters. The literature search was deliberately broad to capture a broad range of considerations that could impact the quality and effectiveness of discharge communications. The final literature review was conducted in October 2021 by IA, and a total of 30 relevant studies were identified (Additional file 1). IA and MF identified key elements that were present in two or more studies and sorted them into an initial coding scheme encompassing 36 key elements. In discussions between IA, EL, and ME, elements were merged if they were considered to refer to the same content. The final matrix underwent revisions until consensus was reached on the minimum required content within each key element (*n* = 11). As a result, this assessment tool will be referred to as *Safe Assessment For Effective Discharge*, SAFE-D.

A deductive content analysis of the discharge letters was conducted using the SAFE-D (Table 1) [34]. The analysis began by thoroughly reading the discharge letters to become familiar with the data and obtain an overview of the texts. Subsequently, the SAFE-D facilitated categorisation, and data from the discharge letters was systematically coded into the matrix (for example see Table 2). The content was reviewed to ensure it met the minimum required content specified by the assessment tool. Next, the coded key elements were transformed into a quantitative variable labelled “SAFE-D score.” The coded key elements that contained the minimum required information were scored 1 point, and if a key element was minimally represented or not present in the discharge letter, the score was 0. By adding up the scores for the key elements, each discharge letter got a score between 0 and 11. The variable, SAFE-D score, was merged with the dataset from the RCT and used in the statistical analyses [28].

**Table 1:** The assessment matrix SAFE-D (Safe Assessment For Effective Discharge) of minimum required content for each key element in discharge letters

Key element	Reason for admission	Progress during care	Diagnosis stated	Medication list	Explanation of medication treatment	Advice about lifestyle or self-management	Follow-up	Contact information	Expected course and complications	Measures in case of deterioration	Patient-friendly discharge letter
Minimum required content	Symptoms that were the reason for the patient being admitted, such as respiratory distress, chest pain or fainting	Descriptions of examinations, treatments and test results	Stating the current diagnosis in an intelligible language; can be a statement that no diagnosis was found that could explain the symptoms	A printed medication list is attached	Any information about the medication treatment, including stating “no changes” or referring to the medication list	Advice about any lifestyle factor, e.g., diet, exercise, tobacco or alcohol, or self-management such as daily weight monitoring	Any information about follow-up; can be the information “no follow-up planned”	Contact information to the ward or to an appropriate clinic (e.g., heart failure clinic)	Predicted disease course and any potential complications, such as symptoms expected to subside, symptoms to watch out for (“red flags”)	Any measure to take in case of deterioration, such as who to contact (e.g., general practitioner, heart failure clinic, emergency department) and the urgency of that contact	No abbreviations except the most common ones (i.e., e.g.), no medical or technical terms if not explained in lay terms

**Table 2:** Example of assessment of minimum required content of key elements in a discharge letter using the SAFE-D

Key element	Minimum required content	Examples of contents in discharge letters
Reason for admission	Symptoms	Respiratory distress, fainting “You came to the hospital because of shortness of breath”
Progress during care	Any description of care provided during the admission	Examinations, treatments, “Ultrasound of the heart shows reduced pumping capacity”
Diagnosis stated	Diagnosis	Exacerbation of COPD^a^ and CHF^b^
Medication list	Printed medication list attached	Medication changes
Explanation of medication treatment	“No changes” or “See attached list”	“Antibiotics for another five days”
Advice about lifestyle and self-management	Advice about lifestyle *or* self-management	Diet recommendation, exercise, Tobacco cessation “It is important that you completely abstain from alcohol going forward”
Follow-up	“No follow-up planned”	“Copy to GP^c”^, “You have a scheduled appointment on Friday next week”
Contact information	Phone number to ward	Pre-populated phone number on discharge letter
Expected course any possible complications	Expected course *or* any complications	Symptoms to watch out for “I don't expect any further symptoms”
Measures in case of deterioration	Any measure to take in case of deterioration	Contact GP, “Emergency care as soon as possible” “Return to hospital in case of deterioration”
Patient-friendly discharge letter	No abbreviations except the most common (i.e., e.g.), medical or technical terms only if explained	“Ultrasound to create images of your heart” rather than “transthoracic echocardiogram”

^a^Chronic obstructive pulmonary disease

^b^Congestive heart failure ^c^General practitioner

### Statistical analyses of the combined dataset

Patient characteristics and discharge letter contents were described with frequencies and percentages or means and standard deviations. Bivariate correlations were used to examine correlations between each key element, the SAFE-D score, and the self-rated quality of care transition (CTM-3), as well as the unplanned readmission rate (30-day and 90-day readmission rates, respectively). All correlations were tested using Pearson’s correlation coefficient with 2-tailed significance.

Time-to-event analyses were carried out to investigate the association between SAFE-D score and time to readmission. First, patients were divided into three groups based on SAFE-D score: below median (< 8, *n* = 31), median (8, *n* = 54), and above median (> 8, *n* = 51) and a Kaplan–Meier analysis was performed. Significant differences between the three groups were tested using a log-rank test.

To identify the association between SAFE-D score and other potential independent predictors of time to readmission at 90 days, a stepwise multivariable Cox proportional hazards model was applied. The model included the following variables: intervention/control group, age at inclusion, sex, education level, living arrangements, income level, country of birth, CTM-3, PAM at baseline, Charlson Comorbidity Index, number of medications and unplanned admission within 180 days before index admission. Stepwise elimination was used in the Cox regression and the results are presented as hazard ratios. The adjusted hazard ratio in the multivariable model compares the readmission rate at any time during follow-up for each explanatory variable when all other explanatory variables are constant [35].

Multiple imputation was used to take advantage of all the data in the Cox proportional hazards model regardless of missing items [36]. In addition to the original dataset, missing data were replaced with five imputed datasets. Variables corresponding to those used in the subsequent analysis were used for multiple imputation [36]. The Cox proportional hazards model was also used without imputed data to verify the multiple imputation model.

The significance level was set to < 0.05 for all tests. All statistical analyses were performed using “SPSS Inc 27.0.1.0”.

## Results

### Patient characteristics

The majority, 133 of 136 patients (97.8%), had multimorbidity (defined as two or more diagnoses) [37, 38]. On average, patients had 5.5 (min 1, max 10) diagnoses at discharge from hospital and a Charlson Comorbidity Index of 6.1 (min 3, max 12). Fifty-six patients (41.2%) had been admitted to inpatient hospital care within 180 days before the index admission. Of all patients, 18.4% were readmitted to the hospital within 30 days and 35.3% were readmitted within 90 days after discharge from index admission (Table 3).

**Table 3:** Patient characteristics at baseline (*n* = 136)

	**Mean (SD)**	**n (%)**
Age (years)	76.2 (8.3)	
Age interval, n (%)		
60–64		12 (8,8)
65–69		20 (14,7)
70–74		24 (17,6)
75–79		35 (25,7)
80–84		19 (14,0)
85–89		20 (14,7)
90–94		6 (4,4)
Sex		
Male		70 (51.5)
Female		66 (48.5)
Education		
Primary school		36 (26.5)
Secondary school		42 (30.9)
University		35 (25.7)
Missing		23 (16.9)
Married/living with partner		
Yes		50 (36.8)
No		63 (46.3)
Missing		23 (16.9)
Monthly income		
< 10,000 SEK		7 (5.1)
10,000–20,000 SEK		51 (37.5)
20,000–50,000 SEK		42 (30.9)
> 50,000 SEK		11 (8.1)
Missing		25 (18.4)
Country of birth		
Sweden		97 (71.3)
Nordic country other than Sweden		8 (5.9)
Outside the Nordic countries		7 (5.1)
Missing		24 (17.6)
Number of diagnoses (ICD-10^a^ codes)	5.5 (2.3)	
Charlson Comorbidity Index	6.1 (1.9)	
Unplanned admission within preceding 180 days^b^		56 (41.2)
Length of stay, index admission (days)	5.8 (4.8)	
Number of medications within preceding 180 days^b^	12.0 (6.2)	
Self-rated quality of care transition CTM-3^c^	77.1 (21.6)	
PAM^d^	55.8 (11.9)	
Readmitted within 30 days of discharge from index admission		25 (18.4)
Readmitted within 90 days of discharge from index admission		48 (35.3)

^a^International Classification of Diseases, 10th revision

^b^Within 180 days before index admission

^c^Care Transition Measure, 3 items

^d^Patient Activation Measure

Patient activation level at the time of discharge was, on average 55.8, which is just above the cut-off for the third activation level (< 55.1). The standard deviation of 11.9 indicates that some patients still perceived a “lack of knowledge and confidence to take action”, but were “beginning to take action in their self-care”.

### Key elements included in discharge letters

**Fig. 1:**
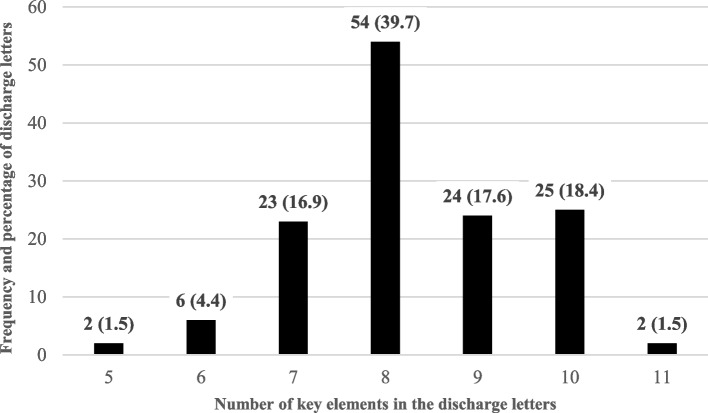
Frequency and percentage of discharge letters by number of key elements, n (%)

The document analysis revealed that all discharge letters contained at least five key elements. On average, 8.3 (min 5, max 11) of the eleven key elements were included in the discharge letters. All eleven key elements were found in two (1.5%) of the discharge letters (Fig. 1).

**Fig. 2:**
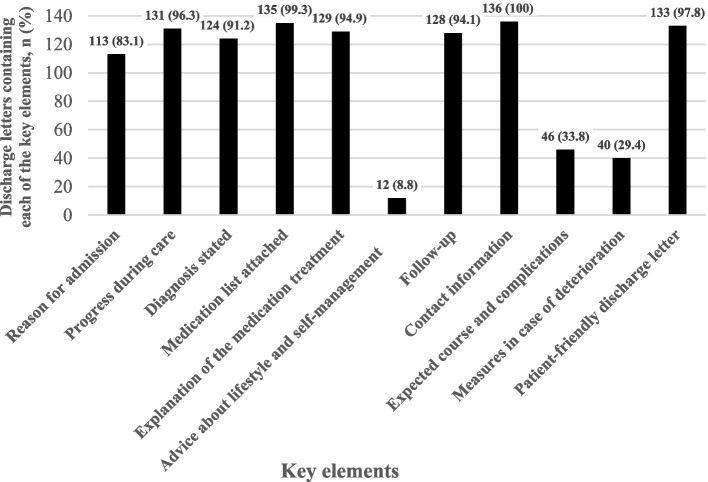
Frequency and percentage of discharge letters containing minimum required information regarding each key element

The most common key elements were “Contact information” (100%), “Medication list” (99.3%) and “Patient-friendly discharge letter” (97.8%). The three least common key elements were “Expected course and complications” (33.8%), “Measures in case of deterioration” (29.4%), and “Advice about lifestyle and self-management” (8.8%). The five remaining key elements were present in between 113 (83.1%) and 131 (96.3%) of the discharge letters (Fig. 2). The discharge letters were reviewed by one of the authors who is a nurse, ensuring they were patient-friendly. This review found that the majority of the letters (133; 97.8%) successfully avoided abbreviations and medical jargon, or clearly explained such terms in language accessible to patients.

### SAFE-D score and key elements in relation to CTM-3

SAFE-D score showed a significant negative correlation with CTM-3 (*p* < 0.05). A statistically significant but weak negative correlation (*r* = -0.24) was also found between the key element “Reason for admission” and CTM-3 (*p* < 0.05). This indicates that more key information in the discharge letter was associated with lower ratings for quality of care transitions (Table 4).

**Table 4:** Correlations between discharge letters content and CTM-3^a^, 30-day or 90-day readmission rate (Pearson’s correlation coefficient)

Key elements/SAFE-D score	CTM-3^a^	30-day readmission rate	90-day readmission rate
Reason for admission	-.24*	.11	.01
Progress during care	-.01	.09	.06
Diagnosis stated	.10	-.05	-.04
Medication list attached	-.00	.04	.06
Explanation of the medication list	-.09	.03	-.04
Advice about lifestyle and self-management	-.12	.05	.15
Follow-up	-.14	-.04	-.14
Contact information	N/A	N/A	N/A
Expected course and complications	-.11	.06	-.01
Measures in case of deterioration	-.16	-.06	-.11
Patient-friendly discharge letter	-.06	-.06	.01
SAFE-D score	-.23*	.05	-.04

^a^Care Transition Measure, 3 items

^*^P < .05

### SAFE-D score and key elements in relation to hospital readmission

The SAFE-D score was not found to correlate significantly with 30-day or 90-day readmission rates, and no correlations were found between individual key elements and hospital readmission within 30 or 90 days (Table 4).

### SAFE-D score and time to readmission

**Fig. 3:**
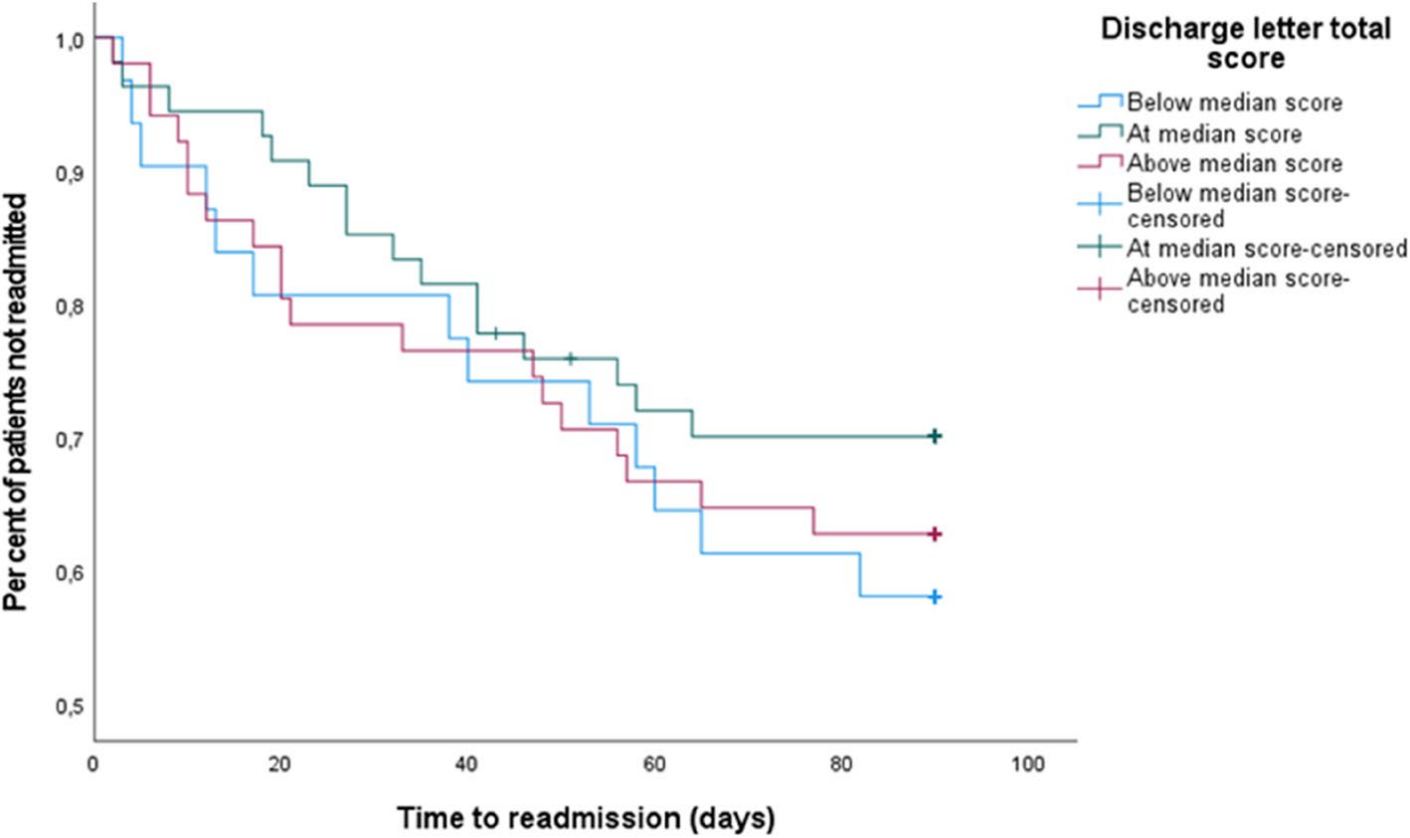
Time to readmission within 90 days among patients below, at and above median SAFE-D score

The estimated mean time to readmission shown in a Kaplan–Meier analysis was 66.8 days for patients with below median SAFE-D score (< 8 key elements), 72.5 days for those with median score (8 key elements) and 67.6 days for those above median score (> 8 key elements). The differences between the Kaplan–Meier survival curves were not significant in a log-rank test, *p* = 0.54 (Fig. 3).

### Cox proportional hazards model to predict time to hospital readmission

The stepwise Cox proportional hazards model had 14 iterations in total. It did not reveal any significant impact of SAFE-D score on time to readmission, neither in the first step nor in the final step, after stepwise elimination of nonsignificant variables. In the first step, the simultaneous contributions of all independent variables on time to readmission were analysed. None of the variables were significantly associated with time to readmission in the first. In the last step, having an unplanned hospital admission within 180 days before the index admission was the only significant predictor of time to readmission at 90 days (hazard ratio 2.14, *p* < 0.01) (Table 5).

**Table 5:** Time to readmission at 90 days. Iterations 1 and 14 in the stepwise Cox proportional hazards model (pooled results)

Iteration number	Variable		Hazard ratio	95% CI^a^	*p-*value
**1**	Intervention group		0.97	0.50–1.89	.93
	Age at inclusion		1.00	0.95–1.05	.94
	Sex	Male	1.00		
		Female	0.95	0.49–1.85	.88
	Education	Primary school (9 years)	1.00		
		Secondary school	0.83	0.34–2.04	.69
		University	0.76	0.27–2.14	.60
	Married/living with partner		0.95	0.38–2.41	.92
	Monthly income	< 10,000 SEK	1.00		
		10,000–20,000 SEK	0.90	0.17–4.74	.89
		20,000–50,000 SEK	0.88	0.16–4.85	.88
		> 50,000	0.87	0.08–8.91	.90
	Country of birth	Sweden	1.00		
		Other Nordic country	1.42	0.39–5.19	.59
		Outside the Nordic countries	2.05	0.59–7.12	.25
	Charlson Comorbidity Index		1.13	0.95–1.34	.18
	Unplanned admission within preceding 180 days^b^		1.67	0.85–3.25	.14
	Length of stay, index admission (days)		1.01	0.94–1.08	.85
	Numbers of medications within preceding 180 days^b^		1.03	0.98–1.09	.26
	CTM-3^c^		1.00	0.99–1.01	.74
	PAM^d^		1.01	0.98–1.05	.46
	SAFE-D score		1.01	0.77–1.33	.93
**14**	Unplanned admission within preceding 180 days^b^		2.14	1.21–3.79	< .01

^a^Confidence interval

^b^Within 180 days before index admission

^c^Care Transition Measure, 3 items

^d^Patient Activation Measure

To verify the multiple imputation model, an additional Cox proportional hazards model was performed without imputed data. This analysis indicated a similar, however not significant result; having an unplanned hospital admission within 180 days before the index admission was the only remaining variable in the final iteration (hazard ratio: 2.11, *p* = 0.08) (Table 6). There were no statistically significant differences in time to readmission between intervention and control patients.

**Table 6:** Time to readmission at 90 days. Iteration 14 (last step) in the stepwise Cox proportional hazards model (model without imputed data)

Iteration number	Variable	Hazard ratio	95% CI^a^	*p-*value
14	Unplanned admission within preceding 180 days^b^	2.11	0.91–4.87	.08

^a^Confidence interval

^b^Within 180 days before index admission

## Discussion

This study aimed to determine the impact of discharge letter content on unplanned 30-day and 90-day hospital readmissions. The study did not show any significant correlation between the number of key elements of the discharge letter and the 30-day or 90-day readmission rates for patients above 60 with chronic obstructive pulmonary disease or congestive heart failure. Additionally, the SAFE-D score did not indicate any association with time to readmission at 90 days, even after adjusting for patient characteristics, such as age, Charlson Comorbidity Index, patient activation at time for discharge, and self-rated quality of care transition. These results suggest that the contents of the discharge letters did not significantly affect the risk of readmission in the studied population.

Care transitions are high-risk processes, particularly for older patients, who have a high prevalence of comorbidity and frailty [10]. In the current study, 98.5% of discharge letters lacked at least one of the key elements identified as necessary for safe care transitions to home in the initial literature review. The three key elements most commonly omitted from discharge letters were “Expected course and complications”, “Measure in case of deterioration” and “Advice about lifestyle and self-management”. Previous studies have identified these elements as particularly important for patients’ self-management after discharge [22, 39], making omission a cause for concern. In this study, we could not determine which of the key elements, if any, were most important for the studied population and the reasons for their omission in the discharge letters. However, there appears to be insufficient information provided in relation to patient needs, suggesting that tailored interventions are needed to support patients' transition from hospital to home. Follow-up in primary care shortly after discharge might provide an opportunity to convey information about any issues that were not sufficiently explained at discharge. The findings of a recent study by Saxena et al*.* [40] suggest that patients who have a follow-up appointment with a primary care physician within seven days of discharge were less likely to be readmitted within 90 days. These results emphasise the importance of improving communication and information transfer during the discharge process. In addition, the study highlights the need for a more comprehensive understanding of how older adults with chronic illnesses obtain the knowledge and support necessary for handling their medication and self-management after being discharged from inpatient hospital care.

There was a negative correlation between SAFE-D score and CTM-3, suggesting that patients who received higher quality discharge letters tended to rate the quality of their care transition as lower. This unexpected finding may imply that factors beyond discharge letters play a significant role in shaping the perceived quality of care during the transition from hospital to home. Even if all key elements were included in a discharge letter, a patient could still feel unprepared to manage their medications and symptoms at home. To strengthen the delivery of integrated healthcare services to patients, a multi-disciplinary team collaboration across healthcare organisations is growing increasingly important [41]. A recent study found that to feel prepared for discharge, patients wanted a follow-up plan, dialogue with healthcare professionals while still in the hospital, and to be involved in decisions during their hospital stay and at admission [42]. To meet these needs, it may be beneficial to involve patients and their families in the discharge process and to collaborate with healthcare professionals in the next stage of care (such as primary care) to ensure that patients receive clear and understandable information after discharge [43].

The discharge process can be experienced as confusing by older patients with complex or multiple diseases [22, 44] who encounter difficulties in finding their way in a healthcare system adapted for single diseases where they have to face multiple specialties and caregivers and navigate among them [45]. Factors other than the contents of the discharge letter might also contribute to readmission. In our study, we found that an unplanned admission within 180 days before the index admission significantly predicted the time to readmission at 90 days. Patient understanding of discharge information was not investigated in this study. Possibly, an understanding of self-management of complex treatment may correlate with illness burden. The mean Charlson Comorbidity Index was relatively high in this patient group (> 6), and the mean PAM scores indicated that some patients still had “lack of knowledge and confidence to take action”, while others were starting to take action for self-management. The previous unplanned admission rate as a predictor of time to readmission may indicate that these patients were fragile and required recurrent acute hospital care. This highlights that patients with chronic illness need not only a discharge letter, but also individual self-management support during care transitions and the first period at home [43, 44].

Previous studies on discharge letter contents and readmission rates have shown ambiguous results. Rodwin et al*.* [46] found that redesigned letter templates, with guiding headings like “documentation of the correct discharge diagnosis”, “information about the admission and treatment”, “disease-specific warning signs” and “issues that require follow-up”, improved the quality of discharge letters more effectively than educational outreach programmes for physicians. Still, the improved quality of discharge letters did not result in a statistically significant change in readmission rates. In a study by Dalley et al*.* [47], standardised discharge letters increased patient satisfaction, though individualised discharge letters are sometimes preferred by patients [48]. However, the overall comprehensibility of discharge letters is crucial to effective and safe communication between the hospital and the general practitioner in a care transition. Ensuring the quality of discharge letters and overcoming barriers such as time limitations and template restrictions is of high priority to ensure patient safety at the point of hospital discharge.

### Strengths and limitations

Given the small sample size and the fact that participants were not specifically recruited for the purpose of evaluating the discharge letter content, the findings of this study should be interpreted with caution. There is a possibility that the observed results may be due to chance, and further research with a larger, targeted sample would be necessary to confirm the study's conclusions and the relative importance of the identified key elements.

One strength of the study is that the identification of key elements were based on the findings of an initial literature review of previous research on the content of discharge letters (for references, see Additional file 1). It was not feasible to rank the relative importance of the eleven key elements identified as important to include in discharge letters; all have been assumed to have the same relevance. Assigning the same importance to all key elements is an approach that has been applied in the reviewed studies. It may be that some elements are of greater importance for patients at discharge than others and that the classification and scoring therefore were suboptimal. It could be argued that for patients to feel more competent in self-management, information about “Advice about lifestyle and self-management” and “Measures in case of deterioration” would be more important than “Progress during care”.

Although patients received discharge letters at the time of discharge, and the majority of discharge letters were judged as patient-friendly, it was not assessed whether they read, comprehended, or followed the advice in the discharge letter. It is important to note that no patients were directly involved in the analysis; instead, the judgments are based on the researcher’s assessment using the SAFE-D. Additionally, the accuracy and completeness of the information in the discharge letters were not evaluated. Other forms of information provision, such as verbal instructions during hospitalisation or at discharge, could also have impacted the patient´s self-management and CTM-3 scores.

## Conclusions

In this study, the number of discharge letter elements did not correspond to a significant change in readmission rates or time to readmission in the intervention group in CHF and COPD patients over 60. Based on these findings, it seems evident that written discharge letters alone are not sufficient to reduce readmission, or for safe care transitions. Furthermore, it is important to note that discharge letters are not the only, nor the most important, aspect of perceived quality in transitional care, and a more comprehensive understanding of channels for communication and information transfer at discharge is needed.

No associations between SAFE-D score and self-rated quality of care transitions, patient activation, readmission rate or time to readmission were found in this study. Key elements essential in promoting a safe care transition, including patient activation after discharge, such as “Measures in case of deterioration” and “Advice about lifestyle and self-management”, were most frequently omitted. Further research is necessary to investigate whether improved quality of discharge letters leads to better patient outcomes. Additionally, understanding how patients use the information within discharge letters and its potential impact on readmission rates warrants exploration beyond the mere contents of the discharge letter.

Additionally, it would be valuable to explore whether standardizing the content of discharge letters could lead to greater consistency and possibly better transitional care. Are there any ongoing efforts within hospitals to standardize these letters, or are they typically composed anew for each patient? These questions point to the need for further studies to determine the optimal approach to discharge communication and its relationship to patient readmission.

### Supplementary Information

Additional file 1. Articles upon which the assessment matrix was based.

## Data Availability

The datasets generated and/or analysed during the current study are not publicly available, as per the Regional Ethical Review Board’s guidelines to protect the privacy of the participants but are available from the corresponding author on reasonable request.
