# Supplementary material for: The impact of discharge letter content on unplanned hospital readmissions within 30 and 90 days in older adults with chronic illness – a mixed methods study
Source: BMC Geriatr. 2024 Jul 10;24:591. doi: 10.1186/s12877-024-05172-1 (PMC11238400; doi:10.1186/s12877-024-05172-1)
Supplement: Supplementary file 1 — Additional file 1. Articles upon which the assessment matrix was based. [file 12877_2024_5172_MOESM1_ESM.pdf]

Additional file 1: Studies identified in the literature review

Abu, H. O., Anatchkova, M. D., Erskine, N. A., Lewis, J., McManus, D. D., Kiefe, C. I., & Santry, H. P. (2018). Are we "missing the big picture" in transitions of care? Perspectives of healthcare providers managing patients with unplanned hospitalization. *Appl Nurs Res*, *44*, 60-66. https://doi.org/10.1016/j.apnr.2018.09.006

Ackermann, S., Heierle, A., Bingisser, M.-B., Hertwig, R., Padiyath, R., Nickel, C. H., Langewitz, W., & Bingisser, R. (2016). Discharge Communication in Patients Presenting to the Emergency Department With Chest Pain: Defining the Ideal Content. *Health Commun*, *31*(5), 557-565. https://doi.org/10.1080/10410236.2014.979115

Borgsteede, S. D., Karapinar-Çarkit, F., Hoffmann, E., Zoer, J., & van den Bemt, P. M. (2011). Information needs about medication according to patients discharged from a general hospital. *Patient Educ Couns*, *83*(1), 22-28. https://doi.org/10.1016/j.pec.2010.05.020

Buckley, B. A., McCarthy, D. M., Forth, V. E., Tanabe, P., Schmidt, M. J., Adams, J. G., & Engel, K. G. (2013). Patient input into the development and enhancement of ED discharge instructions: a focus group study. *J Emerg Nurs*, *39*(6), 553-561. https://doi.org/10.1016/j.jen.2011.12.018

Buurman, B. M., Verhaegh, K. J., Smeulers, M., Vermeulen, H., Geerlings, S. E., Smorenburg, S., & de Rooij, S. E. (2016). Improving handoff communication from hospital to home: the development, implementation and evaluation of a personalized patient discharge

letter. *Int J Qual Health Care*, *28*(3), 384-390. https://doi.org/10.1093/intqhc/mzw046

Cienki, J. J., Guerrera, A. D., Rose Steed, N., Kubo, E. N., & Baumann, B. M. (2013). Impact of an electronic medical record system on emergency department discharge instructions for patients with hypertension. *Postgrad Med*, *125*(5), 59-66. https://doi.org/10.3810/pgm.2013.09.2702

Coleman, E. A., Chugh, A., Williams, M. V., Grigsby, J., Glasheen, J. J., McKenzie, M., & Min, S.-J. (2013). Understanding and execution of discharge instructions. *Am J Med Qual*, *28*(5), 383-391. https://doi.org/10.1177/1062860612472931

Dalley, M. T., Baca, M. J., Raza, C., Boge, L., Edwards, D., Goldszer, R., Cubeddu, L., & Farcy, D. (2020). Does a Standardized Discharge Communication Tool Improve Resident Performance and Overall Patient Satisfaction? *West J Emerg Med*, *22*(1), 52-59. https://doi.org/10.5811/westjem.2020.9.48604

Engel, K. G., Buckley, B. A., Forth, V. E., McCarthy, D. M., Ellison, E. P., Schmidt, M. J., & Adams, J. G. (2012). Patient Understanding of Emergency Department Discharge Instructions: Where Are Knowledge Deficits Greatest? *Acad Emerg Med*, *19*(9), E1035-E1044. https://doi.org/10.1111/j.1553-2712.2012.01425.x

Engel, K. G., Heisler, M., Smith, D. M., Robinson, C. H., Forman, J. H., & Ubel, P. A. (2009). Patient comprehension of emergency department care and instructions: are patients aware of when they do not

understand? *Ann Emerg Med*, *53*(4), 454-e415. https://doi.org/10.1016/j.annemergmed.2008.05.016

Greer, R. C., Liu, Y., Crews, D. C., Jaar, B. G., Rabb, H., & Boulware, L. E. (2016). Hospital discharge communications during care transitions for patients with acute kidney injury: a cross-sectional study. *BMC Health Serv Res*, *16*(1), 449. https://doi.org/10.1186/s12913-016-1697-7

Hansen, L. O., Strater, A., Smith, L., Lee, J., Press, R., Ward, N., Weigelt, J. A., Boling, P., & Williams, M. V. (2011). Hospital discharge documentation and risk of rehospitalisation. *BMJ Qual Saf*, *20*(9), 773-778. https://doi.org/10.1136/bmjqs.2010.048470

Horwitz, L. I., Moriarty, J. P., Chen, C., Fogerty, R. L., Brewster, U. C., Kanade, S., Ziaeian, B., Jenq, G. Y., & Krumholz, H. M. (2013). Quality of discharge practices and patient understanding at an academic medical center. *JAMA Intern Med*, *173*(18), 1715-1722. https://doi.org/10.1001/jamainternmed.2013.9318

Howard-Anderson, J., Busuttil, A., Lonowski, S., Vangala, S., & Afsar-manesh, N. (2016). From discharge to readmission: Understanding the process from the patient perspective. *J Hosp Med*, *11*(6), 407-412. https://doi.org/10.1002/jhm.2560

Jolly, B. T., Scott, J. L., Sanford, S. M., Jolly, B. T., Scott, J. L., & Sanford, S. M. (1995). Simplification of emergency department discharge instructions improves patient comprehension. *Ann Emerg Med*, *26*(4), 443-446. http://search.ebscohost.com.proxy.lnu.se/login.aspx?direct=true&db=

c8h&AN=107382389&site=ehost-live
https://www.annemergmed.com/article/S0196-0644(95)70112-5/fulltext

Karliner, L. S., Auerbach, A., Nápoles, A., Schillinger, D., Nickleach, D., Pérez-Stable, E. J., Karliner, L. S., Auerbach, A., Nápoles, A., Schillinger, D., Nickleach, D., & Pérez-Stable, E. J. (2012). Language barriers and understanding of hospital discharge instructions. *Med Care*, *50*(4), 283-289. https://doi.org/10.1097/MLR.0b013e318249c949

Kergoat, M. J., Latour, J., Julien, I., Plante, M. A., Lebel, P., Mainville, D., Bolduc, A., & Buckland, J. A. (2010). A discharge summary adapted to the frail elderly to ensure transfer of relevant information from the hospital to community settings: a model. *BMC Geriatr*, *10*, 69-69. https://doi.org/10.1186/1471-2318-10-69

LeClair, A. M., Sweeney, M., Yoon, G. H., Leary, J. C., Weingart, S. N., & Freund, K. M. (2019). Patients' Perspectives on Reasons for Unplanned Readmissions. *J Healthc Qual*, *41*(4), 237-242. https://doi.org/10.1097/jhq.0000000000000160

Lin, R., Gallagher, R., Spinaze, M., Najoumian, H., Dennis, C., Clifton-Bligh, R., & Tofler, G. (2014). Effect of a patient-directed discharge letter on patient understanding of their hospitalisation [Journal Article; Randomized Controlled Trial]. *Intern Med J*, *44*(9), 851-857. https://doi.org/10.1111/imj.12482

Regalbuto, R., Maurer, M. S., Chapel, D., Mendez, J., & Shaffer, J. A. (2014). Joint Commission requirements for discharge instructions in

patients with heart failure: is understanding important for preventing readmissions? *J Card Fail*, *20*(9), 641-649. https://doi.org/10.1016/j.cardfail.2014.06.358

Rice, H., Say, R., & Betihavas, V. (2018). The effect of nurse-led education on hospitalisation, readmission, quality of life and cost in adults with heart failure. A systematic review. *Patient Educ Couns*, *101*(3), 363-374. https://doi.org/10.1016/j.pec.2017.10.002

Rodwin, B. A., Bilan, V. P., Gunderson, C. G., & Merchant, N. B. (2021). Improving the Quality of Inpatient Discharge Instructions: an Evaluation and Implementation of Best Practices. *South Med J*, *114*(8), 445-449. https://doi.org/10.14423/SMJ.0000000000001284

Sarzynski, E., Hashmi, H., Subramanian, J., Fitzpatrick, L., Polverento, M., Simmons, M., Brooks, K., & Given, C. (2017). Opportunities to improve clinical summaries for patients at hospital discharge. *BMJ Qual Saf*, *26*(5), 372-380. https://doi.org/10.1136/bmjqs-2015-005201

Schwarz, C. M., Hoffmann, M., Schwarz, P., Kamolz, L.-P., Brunner, G., & Sendlhofer, G. (2019). A systematic literature review and narrative synthesis on the risks of medical discharge letters for patients' safety. *BMC Health Serv Res*, *19*(1), N.PAG-N.PAG. https://doi.org/10.1186/s12913-019-3989-1

Schwarz, C. M., Hoffmann, M., Smolle, C., Eiber, M., Stoiser, B., Pregartner, G., Kamolz, L. P., & Sendlhofer, G. (2021). Structure, content, unsafe abbreviations, and completeness of discharge summaries: a retrospective analysis in a University Hospital in Austria. *J Eval Clin Pract,* 27(6). https://doi.org/10.1111/jep.13533

Shoeb, M., Merel, S. E., Jackson, M. B., & Anawalt, B. D. (2012). "Can we just stop and talk?" Patients value verbal communication about discharge care plans. *J Hosp Med*, *7*(6), 504-507. https://doi.org/10.1002/jhm.1937

Smolle, C., Schwarz, C. M., Hoffmann, M., Kamolz, L. P., Sendlhofer, G., & Brunner, G. (2021). Design and preliminary evaluation of a newly designed patient-friendly discharge letter - a randomized, controlled participant-blind trial [Journal Article; Randomized Controlled Trial]. *BMC Health Serv Res*, *21*(1), 450. https://doi.org/10.1186/s12913-021-06468-3

Vashi, A., & Rhodes, K. V. (2011). "Sign right here and you're good to go": a content analysis of audiotaped emergency department discharge instructions. *Ann Emerg Med*, *57*(4), 315-322.e311. https://doi.org/10.1016/j.annemergmed.2010.08.024

Verhaegh, K. J., Buurman, B. M., Veenboer, G. C., de Rooij, S. E., & Geerlings, S. E. (2014). The implementation of a comprehensive discharge bundle to improve the discharge process: a quasi-experimental study. *Neth J Med*, *72*(6), 318-325.

Weetman, K., Spencer, R., Dale, J., Scott, E., & Schnurr, S. (2021). What makes a "successful" or "unsuccessful" discharge letter? Hospital clinician and General Practitioner assessments of the quality of discharge letters. *BMC Health Serv Res*, *21*(1), 1-16. https://doi.org/10.1186/s12913-021-06345-z
